# Treatment for COVID-19—a cohort study from Northern Italy

**DOI:** 10.1038/s41598-021-00243-4

**Published:** 2021-10-25

**Authors:** Lorenzo Guglielmetti, Daniela Aschieri, Irina Kontsevaya, Francesco Calabrese, Alessandra Donisi, Alberto Faggi, Patrizia Ferrante, Elisa Fronti, Laura Gerna, Maria Cristina Leoni, Franco Paolillo, Giovanna Ratti, Alessandro Ruggieri, Daria Sacchini, Marta Scotti, Caterina Valdatta, Marco Stabile, Gloria Taliani, Mauro Codeluppi

**Affiliations:** 1 Sorbonne Université, INSERM, U1135, Centre d’Immunologie et des Maladies Infectieuses, Cimi-Paris, équipe 13, Paris, France; 2 APHP, Groupe Hospitalier Universitaire Sorbonne Université, Hôpital Pitié-Salpêtrière, Centre National de Référence Des Mycobactéries Et de La Résistance Des Mycobactéries Aux Antituberculeux, Paris, France; 3 Infectious Diseases Unit, Guglielmo da Saliceto Hospital, Piacenza, Italy; 4 Cardiology Unit, Castel San Giovanni Hospital, Piacenza, Italy; 5 Research Center Borstel, Borstel, Germany; 6 German Center for Infection Research, Hamburg-Lübeck-Borstel-Riems, Borstel, Germany; 7 International Health/Infectious Diseases, University of Lübeck, Lübeck, Germany; 8 Migration Health Unit, Primary Health Care Department, Guglielmo da Saliceto Hospital, Piacenza, Italy; 9 Institute for Cross-Disciplinary Physics and Complex Systems IFISC (UIB-CSIC), Campus Universitat Illes Balears, 07122 Palma de Mallorca, Spain; 10 Plastic Surgery Unit, Castel San Giovanni Hospital, Piacenza, Italy; 11 Infectious and Tropical Disease Unit, Department of Translational and Precision Medicine, Sapienza University of Rome, Rome, Italy; 12 Anti-COVID Task Force of the Italian Civil Protection, Rome, Italy

**Keywords:** Epidemiology, Viral infection

## Abstract

Multicentre, retrospective cohort study with multivariable Cox proportional-hazards modelling and survival-time inverse-probability-weighting, evaluating the impact of different treatments on survival of proven COVID-19 patients admitted to two Hospitals in the province of Piacenza, Italy. Use of tocilizumab and of high doses of low molecular weight heparin, but not of antivirals (either alone or in combination), azithromycin, and any corticosteroid, was independently associated with lower mortality. Our results support further clinical evaluation of high doses of low molecular weight heparin and tocilizumab as COVID-19 therapeutics.

## Introduction

Italy was struck by the first epidemic wave of coronavirus disease 2019 (COVID-19) in early 2020^1^. Overall, about 44 000 excess deaths occurred in this 3-month period^2^, largely concentrated in Northern Italy. At that time, virtually no evidence was available on the optimal management of COVID-19. In this study, we aim to evaluate the impact of the medical interventions on survival in hospitalized COVID-19 patients in the province of Piacenza, Italy.

## Methods

A multicentre, retrospective cohort study was performed among patients hospitalized for COVID-19 from February 21st (date of the first reported COVID-19 case) to May 15th, 2020, at two Hospitals in the province of Piacenza: Guglielmo da Saliceto and Castel San Giovanni. Data for consecutive patients were extracted from electronic medical files, cross-checked, and collated in an anonymized database. Data were censored on June 30th, 2020. Adult (18 years and older) confirmed COVID-19 cases with SARS-CoV-2 reverse transcriptase real-time polymerase chain reaction test on nasal/pharyngeal swab^3^ were included. Part of the cohort was described previously^4^. The study was approved by the local Ethics Committee (Area Vasta Emilia Nord), which waived the requirement for informed consent. Continuous data were presented as median and interquartile range (IQR), categorical data as counts and proportions. Low molecular weight heparin was defined as high-dose (HD-LMWH) when given at therapeutic posology (i.e. enoxaparin 6000 international units daily or more) according to drug package inserts. Prescription of treatment (including the choice of the drug and its posology) was heterogeneous in terms of indication as little evidence and no specific recommendations on their use were available. Missing data were handled with multiple imputation using chained equations with 10 imputed datasets. The proportion of missing observations ranged between 0 and 9%. Multivariable Cox proportional-hazards models were used to assess the association of treatment variables with survival, controlling for potential confounders chosen according to univariate results and a priori plausibility. Two models were used: (1) including a variable coding for treatment with any antiviral drug, and (2) including variables coding for the use of hydroxychloroquine, protease inhibitors, and their combination. Variables were included into the model if associated with the outcome at p-value < 0.20 in univariate and if they fulfilled the proportional hazards assumption. Hazard ratios (HR) were reported with 95%-confidence intervals (CI). A sensitivity analysis was performed excluding patients who died at hospital admission or in the two following days. We used survival-time inverse-probability-weighting (IPW) to assess the effect of treatment variables significantly associated with survival in the Cox model. Treatment and censoring models included age, number of comorbidities, symptoms (cough and diarrhoea), respiratory support, and blood tests (total white blood cells and platelets) at hospital admission. Two-sided α < 0.05 was considered statistically significant. Statistical analysis was performed using Stata software version 15.0 (StataCorp).

### Compliance with existing regulations

All methods described in the manuscript were carried out in accordance with relevant guidelines and regulations.

## Results

Overall, 623 patients with a COVID-19 diagnosis were assessed for study participation: out of them, 600 were confirmed COVID-19 cases and were included in the study. Baseline characteristics are provided in Supplementary Tables. At hospital admission, 75% patients required oxygen therapy, 10% required high-flow oxygen therapy or non-invasive ventilation, and 3% required invasive ventilation. Most patients (88%) received antiviral treatment and 71% received at least two antivirals, mostly hydroxychloroquine (79%) and darunavir/cobicistat (54%). In addition, 66% of patients received azithromycin, 44% a corticosteroid, and 5% tocilizumab. Prophylaxis with LMWH was administered to 72% of patients, while 26% received HD-LMWH. As of June 30th, 2020, 355 patients (60%) were discharged, 199 (33%) died, and 46 (7%) were still hospitalized. Full results are reported in Supplementary Tables. Table 1 reports the results of the multivariable Cox proportional-hazards model. Overall, antiviral treatment with any drug, alone or in combination, or treatment with specific drugs (hydroxychloroquine or a protease inhibitor) was not independently associated with increased survival. Similar results were found for treatment with any corticosteroid or azithromycin. Conversely, two treatment variables were associated with increased survival: use of HD-LMWH (HR 0.56; 95% CI 0.39–0.81) and of tocilizumab (HR 0.13; 95% CI 0.02–0.92). These results were confirmed in the sensitivity analysis excluding patients who died early after hospital admission (N = 31; data not shown). According to IPW, the estimated average treatment effect in a population receiving HD-LMWH was an 86% increase (95% CI 27–145%) in survival time relative to a population not receiving it. Conversely, the effect of tocilizumab was not statistically significant (relative average treatment effect: − 1%, 95% CI − 14 to 12%).

**Table 1:** Model-adjusted risk of mortality for medical interventions among COVID-19 patients in multivariable Cox proportional-hazards model. (N = 600).

Variable	Hazard ratio	95% confidence interval	P value
Any antiviral drug*	0.85	0.55–1.29	0.440
Hydroxychloroquine	0.72	0.48–1.08	0.104
Any protease inhibitor^#^	1.17	0.77–1.80	0.457
Combination of at least two antiviral drugs*	0.84	0.61–1.17	0.309
Azithromycin	0.88	0.65–1.19	0.412
Any corticosteroid	0.84	0.72–1.31	0.842
Tocilizumab	0.13	0.02–0.92	0.041
High-dose low molecular weight heparin	0.56	0.39–0.81	0.002

Model adjusted for age, sex, number of comorbidities, patient symptoms (cough or diarrhoea) at hospital admission, level of respiratory support needed at hospital admission, laboratory results (full blood counts of white blood cells, lymphocytes, platelets, and C-reactive protein levels) at hospital admission.

*Including hydroxychloroquine, lopinavir/ritonavir, darunavir/ritonavir, darunavir/cobicistat, or remdesivir.

^**#**^Including lopinavir/ritonavir, darunavir/ritonavir, or darunavir/cobicistat.

## Conclusions

In our study, the administration of antivirals (alone or in combination), azithromycin, and corticosteroids did not lead to increased survival in a cohort of COVID-19 patients. Conversely, the use of HD-LMWH and tocilizumab was independently associated with lower mortality. Our results confirm the lack of mortality reduction in hospitalized COVID-19 patients treated with hydroxychloroquine^5^ or lopinavir/ritonavir^5^. The small number of patients receiving remdesivir in our cohort did not allow to assess its efficacy, which is still uncertain^5,6^. In contrast with recent studies^7^, corticosteroids were not associated with increased survival; this may be partially explained by the heterogeneity in the choice of corticosteroid, its posology, and the timing of treatment in our cohort. Current knowledge on tocilizumab for COVID-19 is highly controversial^8^. In our study, tocilizumab use was associated with lower mortality in survival analysis; however, this finding was not confirmed by IPW. Finally, HD-LMWH use was associated with prolonged survival in our cohort. This supports previous observational evidence that LMWH use may lower COVID-19 mortality^9^, in particular among critically-ill patients and at high doses^10^.

Our study has many limitations: the limited sample size; the observational, retrospective design which implies indication bias for the choice of treatment (including posology of LMWH); and the lack of some relevant variables in our dataset. Robust statistical methods were used to assess the causal relationship between treatment and outcomes: however, the presence of residual, unmeasured confounders cannot be excluded. In conclusion, our results support further study of HD-LMWH and tocilizumab as COVID-19 therapeutics. Randomized controlled trials are needed to assess their role in the management of COVID-19.

## Supplementary Information

Supplementary Information.

## Data Availability

Data may be made available by contacting directly the corresponding author.
