# Supplementary material for: Treatment for COVID-19—a cohort study from Northern Italy
Source: Sci Rep. 2021 Oct 25;11:20964. doi: 10.1038/s41598-021-00243-4 (PMC8545945; doi:10.1038/s41598-021-00243-4)
Supplement: Supplementary file 1 — Supplementary Information. [file 41598_2021_243_MOESM1_ESM.docx]

## Supplementary Materials

## Treatment for COVID-19 – a cohort study from Northern Italy

## Authors:

Lorenzo Guglielmetti, M.D., Ph.D.^1,2,3^ Daniela Aschieri, M.D.^4^ Irina Kontsevaya, Ph.D.^5,6,7^ Francesco Calabrese, M.D.^3^ Alessandra Donisi, M.D.^8^ Alberto Faggi, M.D.^3^ Patrizia Ferrante, M.D.^3,9^ Elisa Fronti, M.D.^3^ Laura Gerna, M.D.^3^ Maria Cristina Leoni, M.D.^3^ Franco Paolillo, M.D.^3^ Giovanna Ratti, M.D.^3^ Alessandro Ruggieri, M.D.^3^ Daria Sacchini, M.D.^3^ Marta Scotti, N.P.^3^ Caterina Valdatta, M.D.^3^ Marco Stabile, M.D.^10^ Gloria Taliani, M.D.^3,11,12^ Mauro Codeluppi, M.D.^3^ for the COVID-Piacenza Group**

** Members of the COVID-Piacenza Group are the following: Mario Barbera, Luigi Cavanna, Andrea Magnacavallo, Carlo Moroni, Massimo Nolli, Roberta Schiavo, Matteo Silva (Piacenza Hospital, Piacenza, Italy); Cristina Maestri, Massimo Piepoli (Castel San Giovanni Hospital, Piacenza, Italy).

## Affiliations:

1. Sorbonne Université, INSERM, U1135, Centre d’Immunologie et des Maladies Infectieuses, Cimi-Paris, équipe 13, Paris, France;
2. APHP, Groupe Hospitalier Universitaire Sorbonne Université, Hôpital Pitié-Salpêtrière, Centre National de Référence des Mycobactéries et de la Résistance des Mycobactéries aux Antituberculeux, Paris, France;
3. Infectious Diseases Unit, Guglielmo da Saliceto Hospital, Piacenza, Italy;
4. Cardiology Unit, Castel San Giovanni Hospital, Piacenza, Italy;
5. Research Center Borstel, Borstel, Germany;
6. German Center for Infection Research, Hamburg-Lübeck-Borstel-Riems, Borstel, Germany;
7. International Health/Infectious Diseases, University of Lübeck, Lübeck, Germany;
8. Migration Health Unit, Primary Health Care Department, Guglielmo da Saliceto Hospital, Piacenza, Italy;
9. Institute for Cross-Disciplinary Physics and Complex Systems IFISC (UIB-CSIC), Campus Universitat Illes Balears, E-07122 Palma de Mallorca, Spain;
10. Plastic Surgery Unit, Castel San Giovanni Hospital, Piacenza, Italy;
11. Infectious and Tropical Disease Unit, Department of Translational and Precision Medicine, Sapienza University of Rome, Rome, Italy;
12. Anti-COVID Task Force of the Italian Civil Protection.

**Corresponding author:**

Lorenzo Guglielmetti

e-mail: lorenzo.guglielmetti@aphp.fr

Laboratoire de Bactériologie-Hygiène, Faculté de Médecine Sorbonne Université, 91 Boulevard de l'hôpital, 75634 Paris Cedex 13, France

Tel: +33 1 40 77 97 56

**Supplementary Table 1.** *Baseline clinical characteristics and comorbidities of 600 hospitalized COVID-19 confirmed cases.*

|  | n (%) |
| --- | --- |
| Demographic characteristics |  |
| Sex, male (N=597) | 415 (70) |
| Age, years, median (IQR) [range] (N=598) | 71 (60-79) [19-102] |
| Clinical presentation at hospital admission |  |
| Fever | 525 (88) |
| Dyspnoea | 324 (54) |
| Cough | 308 (51) |
| Fatigue | 154 (26) |
| Diarrhoea | 83 (14) |
| Myalgia | 43 (7) |
| Time from symptoms to hospital admission, days, median (IQR) | 7 (5-10) |
| Comorbidities |  |
| Diabetes mellitus | 111 (19) |
| *Cardiovascular disease* |  |
| Hypertension | 325 (54) |
| Other cardiovascular disease | 151 (25) |
| *Cerebrovascular disease* | 46 (8) |
| *Pulmonary disease* |  |
| COPD | 58 (10) |
| Bronchial asthma | 12 (2) |
| *Cancer* |  |
| Solid cancer | 51 (9) |
| Onco-hematologic cancer | 27 (5) |
| *Chronic kidney disease* | 30 (5) |
| *Chronic liver disease* | 15 (3) |
| *HIV infection* | 4 (1) |
| *Iatrogenic immunosuppression* | 20 (3) |
| *Number of comorbidities* |  |
| None | 185 (31) |
| One | 151 (25) |
| More than one | 264 (44) |
| Total number, median (IQR) | 1 (0-2) |

IQR = interquartile range; COPD = chronic obstructive pulmonary disease; HIV = human immunodeficiency virus.

**Supplementary Table 2.** *Baseline diagnostic test results of 600 hospitalized proven COVID-19 cases.*

|  | n (%) | N with available results |
| --- | --- | --- |
| Positive PCR for SARS-CoV-2 from nasal swab | 600 (100) | 600 |
| Respiratory support needed at hospital admission |  | 600 |
| No oxygen needed | 68 (11) |  |
| Low-flow oxygen (nasal cannula, Venturi mask, or mask with reservoir) | 452 (75) |  |
| High-flow oxygen or CPAP helmet | 62 (10) |  |
| Invasive ventilation | 18 (3) |  |
| Blood laboratory test, unit (reference range) |  |  |
| *Hematologic* |  |  |
| White blood cells, x10^3^/µl, median (IQR) (4-10) | 7,3 (5,4-9,9) | 594 |
| Haemoglobin, g/dl, median (IQR) (13-17) | 13,3 (12,0-14,6) | 591 |
| Platelets, x10^3^/µl, median (IQR) (150-450) | 206 (154-272) | 594 |
| Lymphocytes, x10^3^/µl, median (IQR) (1,5-4) | 0,89 (0,62-1,26) | 595 |
| *Biochemistry* |  |  |
| Blood creatinine, mg/dl, median (IQR) (0,6-1,2) | 0,98 (0,80-1,28) | 596 |
| Prothrombin time, INR, median (IQR) (0,8-1,1) | 1,29 (1,20-1,40) | 549 |
| C-reactive protein, mg/dl, median (IQR) (0-0,5) | 10,7 (5,4-17,3) | 554 |

PCR = polymerase chain reaction; IQR = interquartile range; CPAP = continuous positive airway pressure; INR = international normalized ratio.

**Supplementary Table 3.** *Treatment, respiratory support received, and outcomes by June 30th, 2020, of 600 confirmed COVID-19 cases.*

|  | n (%) |
| --- | --- |
| Treatment* |  |
| *Antivirals* |  |
| Any antiviral | 526 (88) |
| Combination of more than one antiviral | 426 (71) |
| Hydroxychloroquine | 474 (79) |
| Darunavir/cobicistat | 326 (54) |
| Lopinavir/ritonavir | 200 (33) |
| Remdesivir | 7 (1) |
| *Corticosteroids* |  |
| Any corticosteroid^#^ | 266 (44) |
| *Others* |  |
| Azithromycin | 398 (66) |
| Tocilizumab | 27 (5) |
| Low molecular weight heparin, prophylaxis* | 431 (72) |
| Low molecular weight heparin, high dose** | 157 (26) |
| Total admitted to ICU | 126 (21) |
| Outcomes |  |
| Death | 199 (33) |
| Discharge from hospital (home or rehabilitation facility) | 361 (60) |
| Outcome not assigned | 40 (7) |

IQR = interquartile range; ICU = intensive care unit.

# Including dexamethasone, methylprednisolone, and prednisone given orally or intravenously at different posologies.

* Equivalent to enoxaparin 4000 international units daily or less.

** Equivalent to enoxaparin 6000 international units daily or more.

**STROBE Statement—checklist of items that should be included in reports of observational studies**

|  | Item No. | Recommendation | Page  No. | Relevant text from manuscript | |
| --- | --- | --- | --- | --- | --- |
| **Title and abstract** | 1 | (*a*) Indicate the study’s design with a commonly used term in the title or the abstract | 1 | Title | |
|  |  | (*b*) Provide in the abstract an informative and balanced summary of what was done and what was found |  |  | |
| Introduction | | | |  | |
| Background/rationale | 2 | Explain the scientific background and rationale for the investigation being reported | 6 | Introduction | |
| Objectives | 3 | State specific objectives, including any prespecified hypotheses | 6 | “…we aim to evaluate the impact of these treatments on survival in patients with COVID-19 admitted to two Hospitals in the province of Piacenza, Italy.” | |
| Methods | | | |  | |
| Study design | 4 | Present key elements of study design early in the paper | 6 | “A multicentre, retrospective cohort study was performed…” | |
| Setting | 5 | Describe the setting, locations, and relevant dates, including periods of recruitment, exposure, follow-up, and data collection | 6 |  | |
| Participants | 6 | (*a*) *Cohort study*—Give the eligibility criteria, and the sources and methods of selection of participants. Describe methods of follow-up  *Case-control study*—Give the eligibility criteria, and the sources and methods of case ascertainment and control selection. Give the rationale for the choice of cases and controls  *Cross-sectional study*—Give the eligibility criteria, and the sources and methods of selection of participants | 6 | |  |
|  |  | (*b*) *Cohort study*—For matched studies, give matching criteria and number of exposed and unexposed  *Case-control study*—For matched studies, give matching criteria and the number of controls per case |  |  | |
| Variables | 7 | Clearly define all outcomes, exposures, predictors, potential confounders, and effect modifiers. Give diagnostic criteria, if applicable | 6 |  | |
| Data sources/ measurement | 8* | For each variable of interest, give sources of data and details of methods of assessment (measurement). Describe comparability of assessment methods if there is more than one group | 6 | Data collection | |
| Bias | 9 | Describe any efforts to address potential sources of bias | 7 |  | |
| Study size | 10 | Explain how the study size was arrived at | 6 | “consecutive patients” | |

Continued on next page

| Quantitative variables | 11 | Explain how quantitative variables were handled in the analyses. If applicable, describe which groupings were chosen and why | NA |  |
| --- | --- | --- | --- | --- |
| Statistical methods | 12 | (*a*) Describe all statistical methods, including those used to control for confounding | 7 |  |
|  |  | (*b*) Describe any methods used to examine subgroups and interactions | NA |  |
|  |  | (*c*) Explain how missing data were addressed | 7 |  |
|  |  | (*d*) *Cohort study*—If applicable, explain how loss to follow-up was addressed  *Case-control study*—If applicable, explain how matching of cases and controls was addressed  *Cross-sectional study*—If applicable, describe analytical methods taking account of sampling strategy | NA |  |
|  |  | (*e*) Describe any sensitivity analyses | 7 |  |
| Results | | | | |
| Participants | 13* | (a) Report numbers of individuals at each stage of study—eg numbers potentially eligible, examined for eligibility, confirmed eligible, included in the study, completing follow-up, and analysed | 7, 8 |  |
|  |  | (b) Give reasons for non-participation at each stage | NA |  |
|  |  | (c) Consider use of a flow diagram | Not included |  |
| Descriptive data | 14* | (a) Give characteristics of study participants (eg demographic, clinical, social) and information on exposures and potential confounders | 6-7, Supplementary Tables |  |
|  |  | (b) Indicate number of participants with missing data for each variable of interest | 7, Supplementary Table 2 |  |
|  |  | (c) *Cohort study*—Summarise follow-up time (eg, average and total amount) | NA |  |
| Outcome data | 15* | *Cohort study*—Report numbers of outcome events or summary measures over time | 8 |  |
|  |  | *Case-control study—*Report numbers in each exposure category, or summary measures of exposure |  |  |
|  |  | *Cross-sectional study—*Report numbers of outcome events or summary measures |  |  |
| Main results | 16 | (*a*) Give unadjusted estimates and, if applicable, confounder-adjusted estimates and their precision (eg, 95% confidence interval). Make clear which confounders were adjusted for and why they were included | 8, Table |  |
|  |  | (*b*) Report category boundaries when continuous variables were categorized | NA |  |
|  |  | (*c*) If relevant, consider translating estimates of relative risk into absolute risk for a meaningful time period | NA |  |

Continued on next page

| Other analyses | 17 | Report other analyses done—eg analyses of subgroups and interactions, and sensitivity analyses | 8 |  |
| --- | --- | --- | --- | --- |
| Discussion | | | | |
| Key results | 18 | Summarise key results with reference to study objectives | 9 |  |
| Limitations | 19 | Discuss limitations of the study, taking into account sources of potential bias or imprecision. Discuss both direction and magnitude of any potential bias | 9 |  |
| Interpretation | 20 | Give a cautious overall interpretation of results considering objectives, limitations, multiplicity of analyses, results from similar studies, and other relevant evidence | 9-10 |  |
| Generalisability | 21 | Discuss the generalisability (external validity) of the study results | 9-10 |  |
| Other information | |  | | |
| Funding | 22 | Give the source of funding and the role of the funders for the present study and, if applicable, for the original study on which the present article is based | 4 |  |

*Give information separately for cases and controls in case-control studies and, if applicable, for exposed and unexposed groups in cohort and cross-sectional studies.

**Note:** An Explanation and Elaboration article discusses each checklist item and gives methodological background and published examples of transparent reporting. The STROBE checklist is best used in conjunction with this article (freely available on the Web sites of PLoS Medicine at http://www.plosmedicine.org/, Annals of Internal Medicine at http://www.annals.org/, and Epidemiology at http://www.epidem.com/). Information on the STROBE Initiative is available at www.strobe-statement.org.
